# Expression of concern: Preparation of Y-doped ZrO_2_ coatings on MnO_2_ electrodes and their effect on electrochemical performance for MnO_2_ electrochemical supercapacitors

**DOI:** 10.1039/d2ra90016e

**Published:** 2022-02-17

**Authors:** Laura Fisher

**Affiliations:** Royal Society of Chemistry Thomas Graham House, Science Park, Milton Road Cambridge UK

## Abstract

Expression of concern for ‘Preparation of Y-doped ZrO_2_ coatings on MnO_2_ electrodes and their effect on electrochemical performance for MnO_2_ electrochemical supercapacitors’ by Yuqing Zhang *et al.*, *RSC Adv.*, 2016, **6**, 1750–1759, DOI: 10.1039/C5RA20543C.

The following article ‘Preparation of Y-doped ZrO_2_ coatings on MnO_2_ electrodes and their effect on electrochemical performance for MnO_2_ electrochemical supercapacitors’ has been published in *RSC Advances*.

The SEM in Fig. 6C, which represents Y/ZrO_2_@MnO_2_ particles after 5000 cycles, is a rotated and scaled section of Fig. 6B, which represents Y/ZrO_2_@MnO_2_ particles.

The authors were contacted for comment and asked to provide raw data but have not responded to these concerns. *RSC Advances* is publishing this expression of concern to alert readers to the concerns raised. An expression of concern will continue to be associated with the article until we receive conclusive evidence regarding the reliability of the reported data.

Laura Fisher

11^th^ February 2022

Executive Editor, *RSC Advances*

## Supplementary Material

